# Diverse and variable virus communities in wild plant populations revealed by metagenomic tools

**DOI:** 10.7717/peerj.6140

**Published:** 2019-01-11

**Authors:** Hanna Susi, Denis Filloux, Mikko J. Frilander, Philippe Roumagnac, Anna-Liisa Laine

**Affiliations:** 1 Research Centre for Ecological Change, Organismal and Evolutionary Biology Research Programme, University of Helsinki, Finland; 2 CIRAD, BGPI, Montpellier, France; 3 BGPI, INRA, CIRAD, SupAgro, University Montpellier, Montpellier, France; 4 Institute of Biotechnology, Genome Biology Program, University of Helsinki, Finland

**Keywords:** Betapartitivirus, Caulimovirus, Closterovirus, Metagenomics, Enamovirus, *Plantago lanceolata latent virus*, Pathogens, Epidemiology, Landscape, Small RNA

## Abstract

Wild plant populations may harbour a myriad of unknown viruses. As the majority of research efforts have targeted economically important plant species, the diversity and prevalence of viruses in the wild has remained largely unknown. However, the recent shift towards metagenomics-based sequencing methodologies, especially those targeting small RNAs, is finally enabling virus discovery from wild hosts. Understanding this diversity of potentially pathogenic microbes in the wild can offer insights into the components of natural biodiversity that promotes long-term coexistence between hosts and parasites in nature, and help predict when and where risks of disease emergence are highest. Here, we used small RNA deep sequencing to identify viruses in *Plantago lanceolata* populations, and to understand the variation in their prevalence and distribution across the Åland Islands, South-West Finland. By subsequent design of PCR primers, we screened the five most common viruses from two sets of *P. lanceolata* plants: 164 plants collected from 12 populations irrespective of symptoms, and 90 plants collected from five populations showing conspicuous viral symptoms. In addition to the previously reported species *Plantago lanceolata latent virus* (PlLV), we found four potentially novel virus species belonging to *Caulimovirus, Betapartitivirus, Enamovirus,* and *Closterovirus* genera. Our results show that virus prevalence and diversity varied among the sampled host populations. In six of the virus infected populations only a single virus species was detected, while five of the populations supported between two to five of the studied virus species. In 20% of the infected plants, viruses occurred as coinfections. When the relationship between conspicuous viral symptoms and virus infection was investigated, we found that plants showing symptoms were usually infected (84%), but virus infections were also detected from asymptomatic plants (44%). Jointly, these results reveal a diverse virus community with newly developed tools and protocols that offer exciting opportunities for future studies on the eco-evolutionary dynamics of viruses infecting plants in the wild.

## Introduction

Plants harbor a wide diversity of microorganisms both inside and outside their tissues, and a fraction of this microbial diversity is known or suspected to be pathogenic (Vandenkoornhuyse et al., 2015). Our understanding of the diversity of potentially pathogenic microbes and their impact on both domesticated plants (Bulgarelli et al., 2015) and model organisms, such as *Arabidopsis thaliana* (Horton et al., 2014; Müller et al., 2016) has increased dramatically following advances in sequencing technologies. However, far less is known about the diversity of potentially pathogenic microbes in natural plant populations. Uncovering pathogen diversity in wild plants is non-trivial as this diversity is expected to impact pathogen epidemiology and evolution as well as virulence suffered by the host (Tollenaere, Susi & Laine, 2016). Moreover, diversity of microbes in the wild is expected to be one of the key components of natural biodiversity that promotes long-term coexistence between hosts and parasites in nature (Bartoli et al., 2018). Hence, understanding the diversity of pathogenic microbes in the wild can offer insights into mechanisms that regulate pathogen populations, and thus, can help predict when and where risks of disease emergence are highest.

The ecology and diversity of viruses in wild plant populations has been largely overlooked despite the potential importance of viruses in natural populations and communities (Malmstrom, Melcher & Bosque-Perez, 2011). As pointed out by Malmstrom, Melcher & Bosque-Perez (2011), the under-exploration of virus diversity in wild plant communities is partly due to historical disconnection between plant ecologists and plant pathologists. Plant pathologists have traditionally focused on crop hosts with 77% of recognized plant viruses being initially isolated from cultivated hosts (Wren et al., 2006). In studies of pathogens in wild plant populations, there has been a strong bias towards fungi and bacteria which may be readily identified according to symptoms, via light microscopy or culturing (Burdon & Laine, 2019). Virus detection and identification is challenging as many plant viruses may be asymptomatic, and even when symptoms do occur, they are often impossible to distinguish from those caused by other abiotic or biotic stressors (Agrios, 2005). Hence, traditionally virus detection has relied on electron microscopy or techniques that recognize coat proteins of virus particles, and increasingly on marker-based detection (Agrios, 2005; Hull, 2014). However, uncovering virus diversity in wild plant populations could yield key insights into the eco-evolutionary processes of natural populations for several reasons. First, the limited evidence available suggests that diversity of viruses is high in wild plant communities (Bernardo et al., 2017; Fraile et al., 2017; Rodriguez-Nevado, Montes & Pagan, 2017; Wren et al., 2006). Second, the ecological interaction between the viruses and their host plants range from mutualistic to antagonistic, sometimes in a context-dependent manner (Hamelin et al., 2017). Hence, in addition to being disease causal agents, viruses may contribute significantly to the plant phenotype for example by enhancing stress tolerance (Xu et al., 2008).

As metagenomic tools have become increasingly available and cost-efficient, the paradigm in disease studies has been shifting from one host—one pathogen systems towards understanding multiple pathogens in an ecological community context (Borer, Laine & Seabloom, 2016; Johnson, Roode & Fenton, 2015). To date, the majority of the ecological studies of viruses in wild hosts have focused on individual viruses, and typically those that are also infecting crop species for which there are detection markers available (Malmstrom et al., 2005; Seabloom et al., 2010). These have revealed both high (Prendeville et al., 2012) and variable virus prevalence in the wild (Seabloom et al., 2010; Seabloom et al., 2009; Thapa et al., 2015). More recently, the sequencing of small RNAs from the host has proven a powerful method for uncovering virus diversity (Roossinck, Martin & Roumagnac, 2015). The method is based on the plant resistance mechanism of RNA silencing, where virus specific double-stranded RNA that is generated in most virus infections, is cleaved into 21–24 nucleotide fragments (Dunoyer & Voinnet, 2005). By sequencing these small RNAs, novel viruses from both insects and plants have been discovered (Kreuze et al., 2009; Wu et al., 2010), and we are beginning to gain insight into the distribution of multiple pathogens in wild hosts and across landscape scales (Bernardo et al., 2017; Roossinck, Martin & Roumagnac, 2015). The limitation of small RNA sequencing is failure to detect viruses that either do not trigger silencing responses or that produce silencing suppressors (e.g., persistent viruses; Roossinck, Martin & Roumagnac, 2015).

Here, we characterize virus communities in natural populations of *Plantago lanceolata* in the Åland archipelago, South-West Finland. The study system comprises of approximately 4,000 local populations of *P. lanceolata* that have been monitored for their size annually since 1993 (Ojanen et al., 2013). Since 2001 the host population network has been surveyed for the presence of powdery mildew, *Podopshaera plantaginis*. The survey data coupled with experimental studies have yielded novel insights into eco-evolutionary dynamics that drive population dynamics of both the host and its pathogen (Jousimo et al., 2014; Laine, 2006). Extending studies in the *P. lanceolata* population network to viruses may offer insights into the determinants of potential pathogen communities, and their role in the dynamics of natural plant populations. Our focus is on viruses, as in other geographical areas nearly 40 viruses infecting *P. lanceolata* have been reported (Hammond, 1982), and we have frequently observed symptoms that resemble those caused by viruses in the natural populations in Åland. Moreover, in 2015 we characterized a novel virus species *Plantago lanceolata latent virus* (PlLV), from *P. lanceolata* sampled in Åland (Susi et al., 2017). Here, our aim was to determine the diversity and prevalence of viruses infecting *P. lanceolata*. Hence, we aimed to develop and test protocols that could be used for virus identification from wild populations. For this purpose we used both deep sequencing of small RNAs (Kreuze et al., 2009) and subsequently developed primers for the detection of five common novel viruses identified from the sequence data. Second, this approach was used to investigate how virus infection prevalence varies within and among 12 natural *P. lanceolata* populations. We then investigated whether specific symptoms are associated with the virus species in order to test whether symptoms can be used as a systematic indication of infection in wild plants. Our results show that the five studied viruses (*Plantago lanceolata latent virus*, Plantago latent caulimovirus, Plantago betapartitivirus, Plantago enamovirus and Plantago closterovirus) vary in their prevalence across the *P. lanceolata* populations in the Åland Islands. We also found that the plants showing symptoms were more likely to be infected by one or more virus species than asymptomatic plants (84% infected vs. 44% infected, respectively). Finally, we successfully developed inoculation protocols using sap and aphids for PlLV and using sap for Plantago latent caulimovirus and Plantago enamovirus.

## Material and Methods

### Sampling and nucleic acid extraction

**Figure 1:**
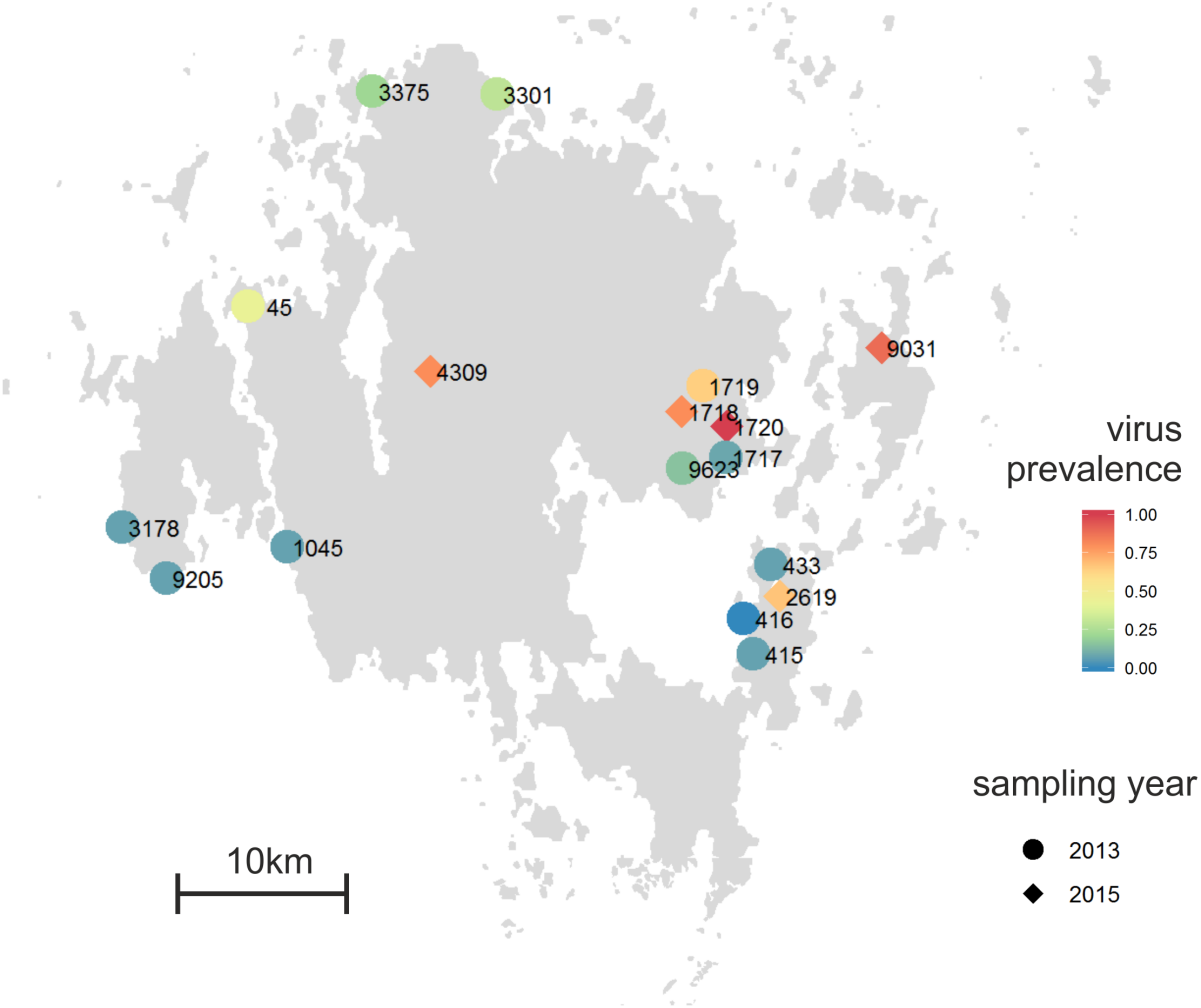
Proportion of individual infected plants across the 12 *Plantago lanceolata* populations in the Åland Islands in 2013 (circles), and five populations in 2015 (diamonds). Viruses were detected from individual plants using (RT-)PCR with specific primers to detect *Plantago lanceolata latent virus,* Plantago latent caulimovirus, Plantago enamovirus, Plantago closterovirus and Plantago betapartitivirus.

*Plantago lanceolata* is a perennial rosette-forming herb with world-wide distribution (Sagar & Harper, 1964). In our study area, the Åland Islands, South-West Finland, it grows typically on dry meadows that are highly fragmented in their distribution (Ojanen et al., 2013). The landscape in the Åland Islands is heterogeneous with the main land-use types being agricultural land, managed mixed forests, largely unmanaged rocky areas, and built areas (Ojanen et al., 2013). In this area, ecological studies on insect herbivores and fungal pathogens have been performed since early 1990’s resulting in a unique database on the size and location of approximately 4,000 *P. lanceolata* populations (Jousimo et al., 2014; Ojanen et al., 2013). The plants for sampling were chosen haphazardly with at least 2 m distance separating the sampled plants. For deep sequencing of the virus-specific small RNAs, we collected leaf samples from 12 *Plantago lanceolata* populations across the Åland Islands in August 2013 (Fig. 1). From each population, samples from 12–14 plants were collected making altogether 164 samples. All samples were immediately frozen in −80 °C and were kept frozen until RNA or DNA was extracted. For detection of RNA viruses, total RNA was separately extracted from each of the 164 samples as described in (Chang, Puryear & Cairney, 1993). For detection of DNA viruses, DNA was separately extracted from the 164 samples using E.Z.N.A. Plant Kit (Omega Biotek, USA).

After RNA extraction, the samples from each population were pooled, resulting in 12 population samples each consisting of 12–14 plant individuals. Small RNA libraries were generated from the pooled samples and sequenced by Fasteris SA (Switzerland) using the method described in (Kreuze et al., 2009). The obtained raw reads were subsequently cleaned to eliminate Illumina adapters and low quality regions (cut-off Phred quality score of 25) using cutadapt (Martin, 2011). De novo assemblies of the cleaned reads were conducted using Velvet (Zerbino & Birney, 2008) and CAP3 (Huang & Madan, 1999) with minimal contig size set at 45 nt.

### Detection of the viruses from the natural populations

In our sequence data, we found virus specific contigs assigned to seven families as well as to unassigned viruses (Table 1; Table S1). Because the similarity of individual matches was low, or contig lengths were short, we used the contigs belonging to same operational taxonomic unit (OTU) to design primers. The threshold for virus identification was that there were enough sequence material matching with published sequences to design detection primers spanning over 1000 nucleotides of the reference virus genome which was not possible for *Endornaviridae* and *Ophioviridae.* Hence, we designed primers for four virus taxa, *Caulimovirus, Betapartitivirus, Enamovirus and Closterovirus*. In addition, we used the PlLV detection primer pair described in Susi & Laine (2017) (Table 2). The RNA samples from each individual plant sampled in 2013 and 2015 were reverse transcribed using iScript™ cDNA Synthesis Kit (Bio-Rad Laboratories, Inc., USA) according to manufacturer’s recommendations. In the PCR reaction, we used 5X Green GoTaq^®^ (Promega Corporation, USA) polymerase and the following thermal cycling conditions: 95 °C for 2 min, 25 cycles of 95 °C (40 s), 50–57 °C (40 s), 72 °C (1 min), and a final extension of 72 °C for 5 min (Table 2). The amplicons were resolved on a 1.2% agarose gel and visualized using Gel Doc XR System (Bio-Rad Laboratories, Inc., USA). The identities of PCR products were confirmed by sequencing samples 3301-124 (enamovirus), 45-92 (caulimovirus), 415-14 (betapartitivirus), and 1719-143 (closterovirus). The specificity of the primers was tested by sequencing the PCR products and they only amplified the sequence specific to the given virus. To validate the primers, we repeated the PCR detections 2–4 times including also coinfected samples. We obtained consistent results across the replicates.

**Table 1:** The number of plant virus associated contigs in pooled samples consisting of 12–14 individual plant samples from single population.

	Population											
	**45**	**415**	**416**	**433**	**1045**	**1717**	**1719**	**3178**	**3301**	**3375**	**9205**	**9623**
Caulimovirus, Caulimoviridae—dsDNA	3	0	0	5	2	1	17	7	1	2	1	0
Betapartitivirus, Partitiviridae—dsRNA	0	4	1	3	6	5	1	0	7	0	0	0
Enamovirus, Luteoviridae—ssRNA (+)	0	0	0	0	0	0	6	0	0	3	0	0
Closterovirus, Closteroviridae—ssRNA (+)	0	0	0	0	0	0	28	2	0	0	0	0
Capulavirus, Geminiviridae—ssDNA	0	0	0	0	0	0	13	10	0	0	0	0
Ophiovirus, Ophioviridae—ssRNA (+)	0	0	0	0	1	0	0	0	0	0	0	1
Endornavirus, Endornaviridae—dsRNA	0	0	0	0	0	0	0	0	0	12	1	0
Unclassified viruses—dsRNA	0	0	0	1	1	1	0	0	0	0	0	0
Unclassified RNA-viruses—ssRNA (−)	0	0	0	0	0	0	1	0	0	0	0	0

**Table 2:** The primers used in detection of the viruses.

Virus	Forward primer 5′–3′	Reverse primer 5′–3′	Product size (nt)	Annealing tm	Approximate sites in the reference genome	Reference accession	Reference
Plantago latent caulimovirus	CAMV_strain_7R TGAAGTTTTTATTATCCGTTCGTACC	CAMV_strain_7F CAAAGCAAATAAAGGAATTACTTGACC	1,089	50	4,404–5,492	AB863200.1	This study
Plantago lanceolata latent virus	CapulaPlantago_1F CAGTCCACACTTCCGCAGTA	CapulaPlantago_2R AACCACACCACCCCAATATC	607	52	1,706–2,313	KT214390.1	Susi et al. (2017)
Plantago betapartitivirus	Hop_1F TCCGTCCTGTTTATGCTGTTGA	Hop_4R TCTTGCAGACATAGTGTGAGGC	974	53	1,136–2,110	NC_021098.1	This study
Plantago enamovirus	Polero_1F GGCTGGCCAAAGAAGGGG	Polero_4 R GCCAGGTTAGTCGACGTGCTCT	929	57	–	–	This study
Plantago closterovirus	Clostero_2F GATTTACCCCAGAACTGTTGGGTG	Clostero_9014R CTAACTTCTTCAGTTAAAGCGCGAGAA	790	50	–	–	This study

We used translated sequences of the obtained sequences in a BlastX search. To understand the phylogenetic relationships, we used 21–46 closest matches of unique sequences obtained from NCBI database for phylogenetic analyses. Sequences were aligned with MUSCLE (Edgar, 2004) implemented in MEGA7 (with default settings) (Kumar, Stecher & Tamura, 2016). A neighbour-joining phylogenetic tree was constructed using MEGA7 (with default settings) and 1,000 bootstrap replicates were used to test the support of branches. Branches with less than 50% bootstrap support were collapsed using TreeGraph2 (Stover & Muller, 2010).

### Sampling of symptomatic plants

To test whether the conspicuous viral symptoms observed in *P. lanceolata* plants in the field indicate virus infection, we collected 90 plants showing typical conspicuous viral symptoms: yellowing, curliness, redness, and necrotic lesions. The samples were collected from five populations, 1718 (9 plants), 1720 (25 plants), 2619 (9 plants), 4309 (10 plants) and 9031 (37 plants) in June 2015 (Fig. 1). As some of the populations sampled in 2015 (1717 and 1719) were mowed and thus unsuitable for sampling, we sampled two adjacent populations (1718 and 1720). The other populations were chosen to represent spatially distant populations. For comparison, we also collected five non-symptomatic plants from each population. In order to understand whether plants showing typical conspicuous viral symptoms were infected by the five viruses studied, (RT-)PCR detections were performed on samples collected from symptomatic and asymptomatic plants.

To test whether virus infections are associated with specific types of visual symptom, we performed analyses as Generalized Linear Models in SAS 9.1 PROC GLIMMIX (SAS Institute Inc., Cary, NC). First, we analysed the association between infection status and visible symptoms for each virus species separately. In each analysis infection status by the target virus (0 = no infection; 1 = infection) was defined as a binary response variable and visual symptoms (redness, yellowing, curliness, and necrotic spots) were defined as categorical explanatory variables. We then analysed whether infection status (0/1) by any of the five tested viruses was associated with the visual symptoms defining the model variables as in the previous analyses. In these analyses, the whole data set of 90 symptomatic plants was used. In these analyses, the sample size was not sufficient to use population as explanatory or random factor.

### Statistical analysis on virus prevalence in populations

In order to understand how infection of individual host plants depends on the virus species and population, we fitted a Generalized Linear Model in SAS 9.1 PROC MIXED (SAS Institute Inc., Cary, NC). Virus infection status of the plant (0 = not infected; 1 = infected) was defined as a binomial response variable with a logit link function, and virus species and host population were used as explanatory class variables in repeated measures model. Plant individual was used as the subject of repeated measures.

To understand how virus species richness differs among the populations, we calculated the Shannon (Shannon & Weaver, 1949) and Simpson diversity indices (Simpson, 1949) in R (R Development Core Team, 2014) using the Vegan package (Oksanen et al., 2018). The number of infected plants by the five viruses in each population was used as data in the analysis.

### Transmission tests for the viruses

To gain insight on transmission ecology and to develop an inoculation method for the study of the five viruses, we tested three different transmission methods: sap inoculation with carborundum powder, sap inoculation using syringe, and aphid (*Dysaphis plantaginea*) transmission. *Dysaphis plantaginea* is a specialist herbivore on *P. lanceolata* and apple (*Malus domestica*) that occurs in the study are in the Åland Islands. In addition to using *P. lanceolata* as virus maintenance plant, we tested inoculation to three plant species commonly used in virus maintenance, *Nicotiana benthamiana, Chenopodium quinoa*, and *Chenopodium amaranticolor,* using the two sap inoculation methods. All virus infected plant and aphid material was collected from the Åland Islands in July 2017 and the plant material was tested with the detection primers as described above. First, we tested whether PlLV can be transmitted by aphids in an inoculation study where a colony of five aphids was first starved for 24 h, then allowed to feed on PlLV infected *P. lanceolata* leaf for 24 h acquisition access period (AAP) followed by 2 h starvation period after which they were placed on uninfected *P. lanceolata* plants for a 2-day inoculation access period (IAP). All *P. lanceolata* individuals used were cloned from mother plants that tested negative for the viruses used in the experiment. In addition, we used (RT-)PCR to verify that the plants tested negative for infection prior to inoculation. DNA samples were taken 14 and 20 days post inoculation (DPI) and the infection was detected using PlLV specific primers as described above. We then performed a set of inoculations with the five viruses using sap from infected plants in 0.02 M phosphate buffer (pH 7.4). Finally, we used the successfully infected plants from the sap and carborundum experiment to test sap inoculation with syringe. In this experiment, the same buffer was used and the leaves of the receiving plants were first wounded with a sterile scalpel and then injected with 100 µL sap and buffer. In this experiment, *P. lanceolata, C. quinoa, C. amaranticolor* and *N. benthamiana* were used as receiving plants (Table 3). Altogether 150 plants were used in the inoculations, described in Table 3, and grown in insect-proof growth chambers at University of Helsinki. From all plants, a leaf sample was taken at 14 or 21 DPI, when virus titre was expected to have reached detectable levels (Agrios, 2005). Subsequently, DNA and RNA were extracted from the plants, and the samples were analysed using PCR or reverse transcription (RT) PCR. The plants were checked for possible symptoms at 14 and 21 DPI.

**Table 3:** Inoculation success of viruses using different methods. The number of inoculations using each method and virus combination is shown in parenthesis.

Inoculation method	PlLV	Plantago latent caulimovirus	Plantago betapartitivirus	Plantago enamovirus	Plantago closterovirus
Aphids to *P. lanceolata*	20% (10)	NA	NA	NA	NA
Sap and carborundum to *P. lanceolata*	30% (20)	0% (8)	0%	0%(4)	0% (4)
Sap and carborundum to *C. quinoa*	100% (19)	33% (3)	0%	25% (4)	0%
Sap and carborundum to *C. amaranticolor*	0% (3)	33% (3)	0%	0%	0%
Sap to *P. lanceolata* (syringe)	40% (5)	0% (12)	NA	0% (4)	NA
Sap to *C. quinoa* (syringe)	90% (10)	0% (12)	NA	0% (3)	NA
Sap to *N. benthamiana* (syringe, from *C. amaranticolor*)	0% (12)	33% (3)	NA	0% (3)	NA

## Results

### Discovery of plant viruses using small RNA-based metagenomics approach

The small RNA sequencing resulted in 10–21 million high quality reads per sample. The reads were assembled into 638 762 contigs ranging from 45 to 5,410 nucleotides (mean 52 nt) using *de novo* assembly. The identities of the contigs were analyzed using BlastX and altogether 147 plant virus-associated contigs were found (Table S1), representing seven virus families and unassigned viruses (Table 1). The mean length of the virus specific contigs was 148, the shortest being 56 nt and the longest 1,837 nt. When we compared the abundance of virus specific contigs in the 12 plant populations, we found that in population 1719 both the number of contigs (66 contigs; 45% of all virus specific contigs) and the number of identified viruses was highest, including PlLV (*Geminiviridae*) and all four putative novel viruses belonging to the *Caulimoviridae, Partitiviridae, Luteoviridae* and *Closteroviridae* families (see below). *Ophioviridae-* related contigs were found in populations 1045 and 9623, and *Endornaviridae-* related contigs in populations 3375 and 9205.

**Figure 2:**
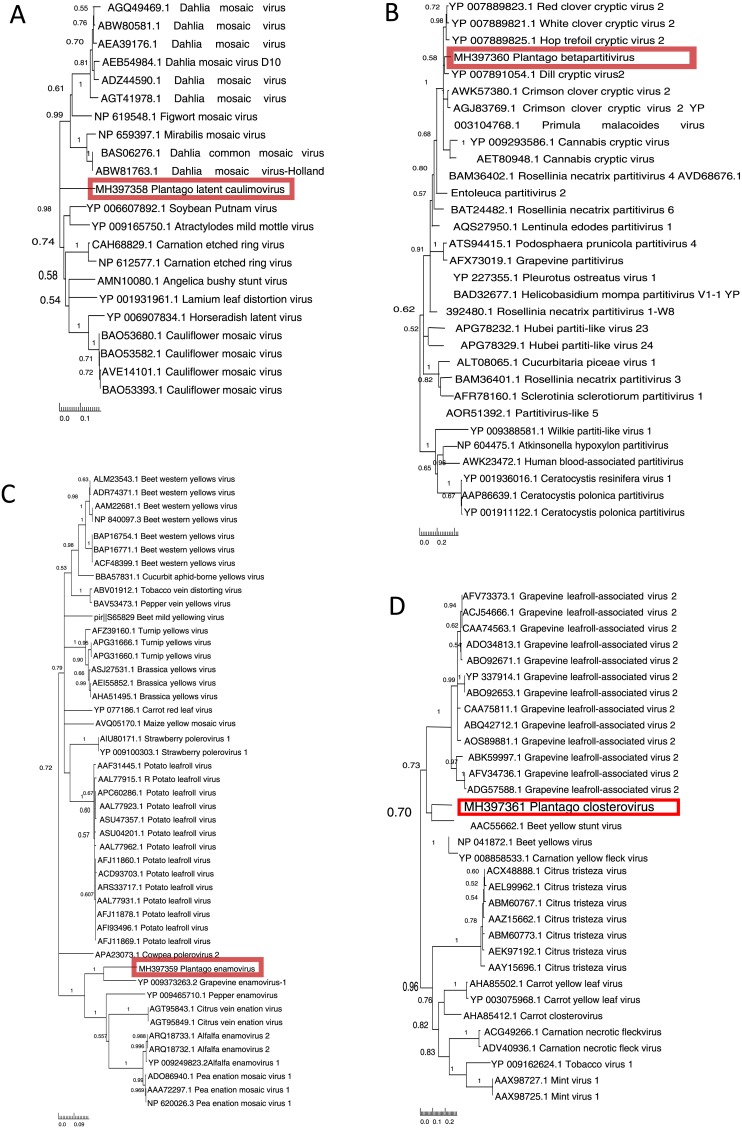
Phylogenetic relationships of the putative novel viruses and related viruses. Translated sequences obtained based on PCR products of the putative novel viruses of *Plantago lanceolata* (highlighted with red) were aligned with MUSCLE implemented in MEGA7 (with default settings). A neighbour-joining phylogenetic tree was constructed using MEGA7 (with default settings), and 1,000 bootstrap replicates were used to test the support of branches. Branches with less than 50% bootstrap support were collapsed using TreeGraph2. Numbers represent support values for particular branches. (A) Plantago latent caulimovirus sequence aligned with 21 polymerase polyprotein sequences retrieved from databases. (B) Plantago betapartitivirus sequence aligned with 30 RNA-dependent RNA polymerase sequences retrieved from databases. (C) Plantago enamovirus sequence aligned with 46 RNA-dependent RNA polymerase sequences retrieved from databases. (D) Plantago closterovirus sequence aligned with 31 heat shock protein sequences retrieved from databases.

### Novel plant viruses identified in *P. lanceolata*

#### Plantago latent caulimovirus

We sequenced the obtained *Caulimoviridae*-related PCR product and used the translated 331 amino acid sequence in a Blast search. We found the closest hit with polyprotein of *Carnation etched ring virus,* genus *Caulimovirus*, (69% similarity, 50% coverage, *e*-value 3e−159). The amino acid sequence was further used to determine phylogenetic relationships with other unique caulimovirus sequences (Fig. 2A). The result showed that while the virus recovered from *P. lanceolata*, hereafter referred to as Plantago latent caulimovirus, had the closest similarity with *Carnation etched ring virus*, it was clustered separately within the known *Caulimovirus* genus (Fig. 2A), suggesting that it is potentially a new species of the *Caulimovirus* genus.

#### Plantago Betapartitivirus

We sequenced the obtained *Partitiviridae*-related RT-PCR product and by using a Blast search, the translated amino acid sequence (263 aa) showed a high degree of similarity to the RdRp of *Dill cryptic virus 2*, genus *Betapartitivirus*, (88% similarity, 35% coverage, *e*-value 1e−164). We analysed its phylogenetic relationship with 22 unique sequences of the known species of the *Partitiviridae* family. The analysis showed that the putative novel betapartitivirus amplified from *P. lanceolata*, hereafter referred to as Plantago betapartitivirus, is nested within the clade composed by eight plant-infecting betapartitiviruses (Lesker, Rabenstein & Maiss, 2013) that clusters separately from the fungus infecting betapartitiviruses (Fig. 2B).

#### Plantago enamovirus

We sequenced the obtained *Luteoviridae*-related PCR product that was translated to 295 amino acids and showed highest similarity (69% similarity, 27% coverage, *e*-value 2e−135) to the RdRp of *Grapevine enamovirus 1* (genus *Enamovirus*) in a Blast search. Pairwise amino acid sequence identity calculation revealed that the 295 amino acids sequence had 49–69%, 49–60% and 49–50% identities with members of the *Enamovirus*, *Polerovirus* and *Luteovirus* genera, respectively. Finally, the phylogenetic analysis revealed that the 295 amino acids sequence branched within the *Enamovirus* genus (Fig. 2C), which suggests that this partial RdRp sequence is likely derived from a novel member of the *Enamovirus* genus, that is hereafter referred to as Plantago enamovirus.

#### Plantago closterovirus

We sequenced the RT-PCR obtained *Closteroviridae*-related PCR product and it was translated to 202 amino acids. In a Blast search it had closest match with heat shock protein 70 (HSP70) of *Beet yellow stunt virus*, genus *Closterovirus* (64% similarity, 38% coverage, *e*-value 2e−86). To understand the phylogenetic relationships of the virus, we analysed the partial HSP70 amino acid sequence together with the other known unique closterovirus sequences. The partial HSP70 sequence we obtained from *P. lanceolata* clustered together with members of the *Closterovirus* genus (Fig. 2D) but was only distantly related to them suggesting that the sequence is likely derived from a novel member of the *Closterovirus* genus, that is hereafter referred to as Plantago closterovirus.

### Distribution of the viruses in the natural populations

**Figure 3:**
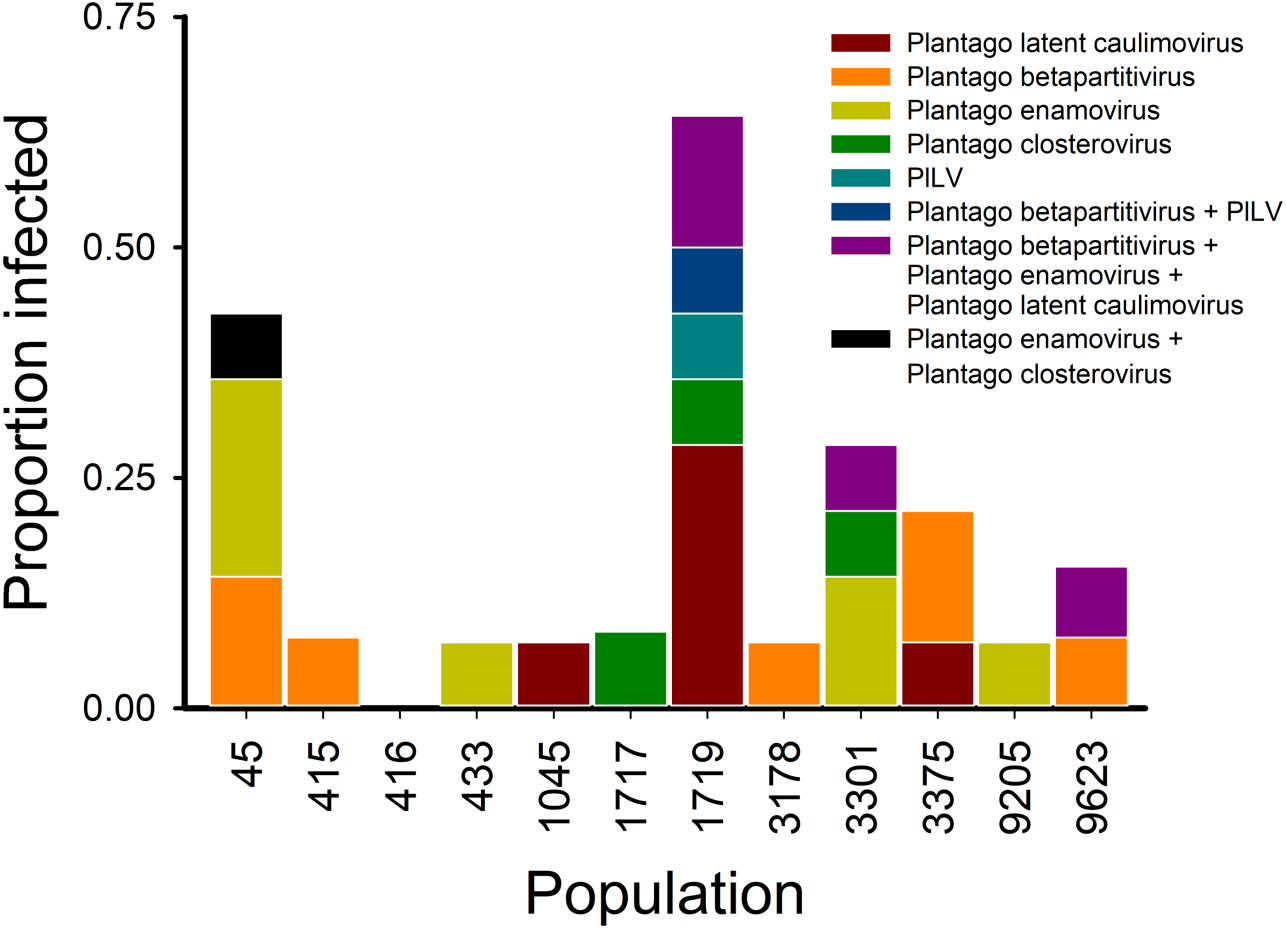
Virus communities (RT-)PCR detected in 12 *Plantago lanceolata* populations in the Åland Islands in 2013. Each bar represents the proportion of individual plants infected by the viruses singly or in coinfection in the population. The colours indicate the prevalence of the studied viruses singly or in coinfection in individual plants.

Using the virus specific primers, we discovered that of the 164 tested plants in 2013, 18.2% were infected by one or more viruses. There was variation among the populations in the proportion of infected individuals (Fig. 1). We found virus-infected plants in all but one of the 12 populations, with the percentage of virus-infected plants varying between 7–64% in the sampled plants (Figs. 1 and 3). When we analysed the factors explaining variation in infection at the individual plant level, we found that the population explained significantly infection prevalence (*d*.*f*. = 11, 153; *F* = 2.04; *P* < 0.0001; Fig. 1), while the virus species did not differ significantly (*d*.*f*. = 4, 656; *F* = 2.04; *P* = 0.0872). The population with highest infection prevalence (1719) was also the only population where all five species were detected. In six of the virus infected populations, only one virus species was detected, while five of the populations supported between two to five of the studied virus species. Across populations, the frequencies of the five virus species varied (Fig. 3). The most abundant virus was Plantago enamovirus which was found in 50% of the populations, and in 7.3% of all sampled plants, whereas PlLV was the rarest virus being detected only in a single population and in 1.2% plants (Fig. 3). (RT-)PCR detections also showed that in 20% of the infected plants, viruses occurred as coinfections (Fig. 3). Plantago enamovirus—Plantago closterovirus coinfection was detected in 13.4% of the infected plants, and Plantago betapartitivirus—PlLV coinfection was detected in 3.3% of the infected plants. Coinfection of three virus species, Plantago betapartitivirus, Plantago enamovirus and Plantago latent caulimovirus was detected in 3.3% of the infected plants. When we analysed the diversity of the virus communities, we found that according to both Shannon and Simpson’s indices, population 45 was the most diverse (Table 4). Overall, there was variation among the populations in their diversity indices (Table 4).

**Table 4:** Prevalence of viruses and richness of the virus communities in *Plantago lanceolata* populations sampled in 2013 in the Åland Islands. (RT-)PCR Virus detections performed on samples collected from 12 wild populations of *Plantago lanceolata*. Shannon diversity and Simpson diversity of the populations are shown.

**Population**	**Sampled plants**	**PlLV**	**Plantago caulimovirus**	**Plantago betapartitivirus**	**Plantago enamovirus**	**Plantago closterovirus**	**Shannon**	**Simpson**	
45	14	0	1	3	4	0	0.571	0.270	
415	14	0	0	1	0	0	0.017	0.005	
416	14	0	0	0	0	0	0.000	0.000	
433	14	0	0	0	1	0	0.016	0.005	
1045	14	0	1	0	0	0	0.008	0.002	
1717	12	0	0	0	0	1	0.005	0.001	
1719	14	2	4	1	2	3	0.052	0.014	
3178	14	0	0	1	0	0	0.003	0.001	
3301	14	0	0	0	3	2	0.012	0.003	
3375	14	0	1	2	0	0	0.008	0.002	
9205	14	0	0	0	1	0	0.001	0.000	
9623	13	0	0	1	1	1	0.003	0.001	

### Virus infection prevalence in plants showing conspicuous viral symptoms

**Figure 4:**
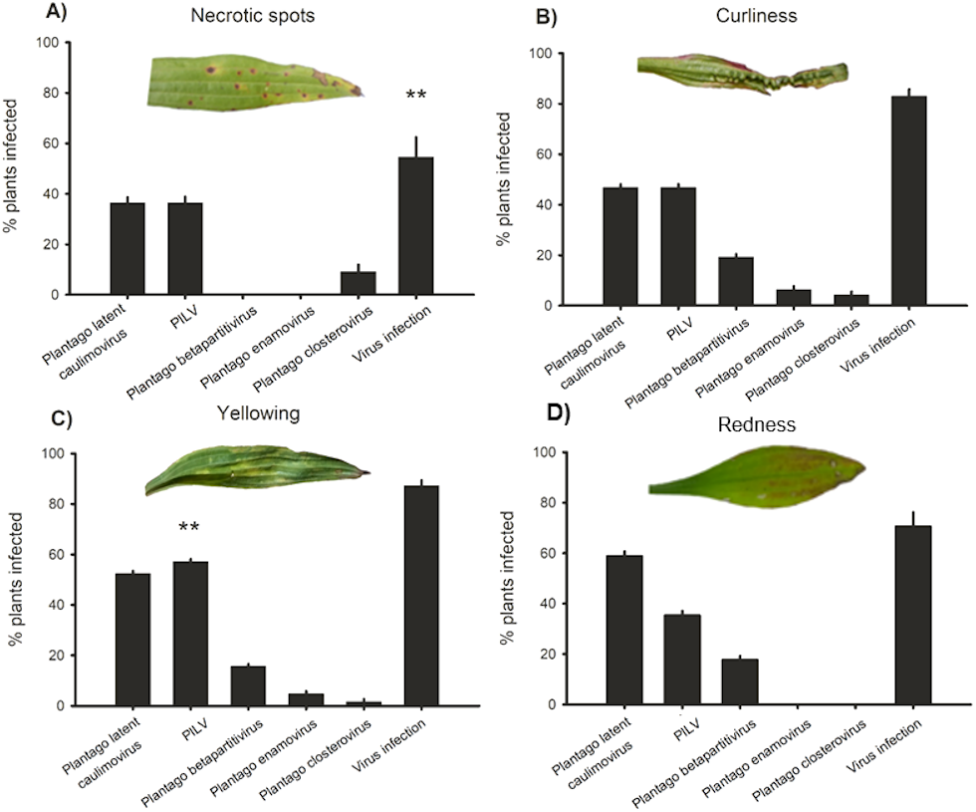
Virus prevalence in symptomatic plants. Positive detections of Plantago latent caulimovirus, *Plantago lanceolata latent virus* (PlLV), Plantago betapartitivirus, Plantago enamovirus, Plantago closterovirus, and infection by any of the viruses alone or in combination in *Plantago lanceolata* showing typical virotic symptoms: (A) necrotic spots, (B) curliness, (C) yellowing, and (D) redness.

In order to understand whether plants showing typical conspicuous viral symptoms were infected by the five viruses studied, (RT-)PCR detections were performed on samples collected from symptomatic plants. We classified the symptoms into four categories: yellowing, redness, necrotic lesions, and curliness. Out of all symptomatic plants collected from the wild, 84% were infected by one or more viruses while in non-symptomatic plants infection prevalence was 44% (Fig. 4). The plants displaying yellowing symptoms were most commonly infected (87% infected plants; Fig. 4). When we looked at the infection prevalence of each virus separately we found a statistically significant association between yellowing and PlLV prevalence (Table 5; Fig. 4).

**Table 5:** Differences of virus infection prevalence in plants showing different virotic symptoms analysed with generalized linear models. Virus infection (single or coinfection of any viruses), infection by Plantago latent caulimovirus, Plantago betapartitivirus, Plantago enamovirus, Plantago closterovirus and PlLV were analysed separately. Statistically significant results (*P* < 0.05) are shown in bold.

		Plantago latent caulimovirus		PlLV		Plantago betapartiti virus		Plantago enamovirus		Plantago closterovirus		Virus infection	
Source	***D.F.***	***F***	***P***	***F***	***P***	***F***	***P***	***F***	***P***	***F***	***P***	***F***	***P***
Redness	1, 85	0.01	0.9123	0.21	0.6504	0.21	0.6499	0	0.9771	0	0.9736	2.52	0.1161
Yellowing	1, 85	0.3	0.5844	**9.6**	**0.0026**	1.13	0.2904	0.25	0.6206	0.13	0.7213	1.47	0.2287
Curliness	1, 85	1.11	0.2946	1.2	0.2768	0.15	0.6954	0.84	0.3628	0.23	0.6358	0.03	0.8698
Necrotic spots	1, 85	1.36	0.2461	1.47	0.2294	0	0.976	0	0.985	1.35	0.2489	**7.89**	**0.0062**

### Transmission of the viruses

We tested three different transmission methods for the viruses: aphid transmission, sap transmission with carborundum and sap transmission with a syringe. We found that PlLV can be transmitted by aphid *D. plantaginea* with an inoculation success rate of 20%. When we tested different transmission methods on *P. lanceolata* and three commonly used virus maintenance plants *C. quinoa, C. amaranticolor* and *N. benthamiana,* we found that PlLV was transmitted successfully with carborundum to *C. quinoa* as 100% of inoculated plants became infected, but the success in other inoculation methods and viruses was lower (0–90% plants infected; Table 3). In addition to PlLV, we were able to develop transmission method for Plantago latent caulimovirus using carborundum to *C. quinoa* (33% plants infected) and *C. amaranticolor* (33% plants infected) and using syringe *to N. benthamiana* (33% plants infected) as well as for Plantago enamovirus to *C. quinoa* using carborundum (25% plants infected; Table 3). *Chenopodium quinoa* proved to be the most amenable plant in the inoculations to maintain viruses. When the plants were checked for viral symptoms at 14 and 21 DPI they did not show any conspicuous symptoms. The inoculations done with Plantago betapartitivirus and Plantago closterovirus did not yield positive detections.

## Discussion

Here, we used small RNA sequencing approach to detect novel viruses in wild populations of *P. lanceolata*, and subsequently addressed the question of how virus prevalence and diversity is distributed among the populations. We designed detection primers for four viruses sharing similarities with members of the *Caulimovirus, Enamovirus, Betapartitivirus*, and *Closterovirus* genera, and uncovered diverse virus communities across the populations studied. These putative novel viruses had relatively low similarities with previously characterized viruses, suggesting that they may be novel viruses. Interestingly, three of the detected viruses associated to the *Caulimovirus, Closterovirus* and *Luteovirus* genera belong to families that have wide host ranges and are commonly reported infecting crops. This suggests that through its role as a host to these viruses, *P. lanceolata* may have the potential to mediate epidemiology of agricultural pathogens. In contrast, new members of one of the virus taxa, *Partitiviridae*, have been recently characterized in both fungi and wild plant hosts (Nibert et al., 2014). Previous studies on betapartitiviruses have suggested that they often occur asymptomatically in wild hosts (Lesker, Rabenstein & Maiss, 2013), and our detection results provide new information on their prevalence in wild populations. Members of family *Partitiviridae* have been suggested to be transmitted either by fungi (Nerva et al., 2017) or through the germ line (Roossinck, 2010). We also came across the common hindrance in *de novo* virus identification; the contigs assembled covered only short fractions of the published genomes (François et al., 2018). Therefore, it was not possible to design primers to amplify the fragments that would span the whole genomes of the viruses. To fully characterize these novel viruses sequencing the full length genomes would be needed.

We surveyed variation in virus infection prevalence within 12 *P. lanceolata* populations and found variation in infection prevalence among the populations. This is in line with other studies that have investigated the distribution of viruses in wild plant populations (Biddle, Linde & Godfree, 2012; Fraile et al., 2017; Pagán et al., 2012; Rodriguez-Nevado, Montes & Pagan, 2017). The diversity indices we used to characterize the local virus communities showed that it is not only virus infection prevalence that varies among the populations, but also the diversity of the local virus communities. In our data, Plantago enamovirus was the most commonly detected virus species. Interestingly, viruses were frequently detected in coinfections consisting of 2–3 virus species among Plantago enamovirus and Plantago closterovirus, Plantago betapartitivirus and PlLV as well as Plantago betapartitivirus, Plantago enamovirus and Plantago latent caulimovirus combinations. To date, studies have characterized novel viruses mainly in cultivated crops (Roossinck, Martin & Roumagnac, 2015) while very few studies have aimed to report novel viruses in wild hosts (Bernardo et al., 2017; Kamitani et al., 2016). An exciting avenue of future research will be to identify factors explaining the differences we observe here in virus communities among the populations. Resistance against viruses in *P. lanceolata* is currently unexplored, but significant genetic variation in resistance against fungal pathogens (De Nooji & van der Aa, 1987; Susi & Laine, 2015; Susi & Laine, 2017) and herbivores (Adler, Schmitt & Bowers, 1995; Barton, 2007) has been reported. Other factors driving the distribution patterns may include differences in pathogen genetic diversity (Rodriguez-Nevado, Montes & Pagan, 2017), vector dynamics (Borer et al., 2010; Hall et al., 2010), the abiotic environment (Seabloom et al., 2010) and spill over from crops (Bell & Tylianakis, 2016; Bernardo et al., 2017).

Virus infections in wild populations are often symptomless (Prendeville et al., 2012), and even when symptoms are found it is difficult to distinguish virus symptoms from those caused by other abiotic or biotic stressors. We aimed to test how well different conspicuous viral symptoms predict infection by one or more of the viruses studied in the wild populations of *P. lanceolata*. We found that plants supporting typical conspicuous viral symptoms in the field were also those likely to be infected by one or more of the five studied viruses. Out of all symptomatic plants, 84% were infected by one or more viruses in contrast to 18% infection when sampling was done without regard to symptoms. Interestingly, 44% of the plants that were symptomless in 2015 sampling were infected by viruses. This highlights the importance of targeting also asymptomatic plants when aiming to unravel the full diversity of virus communities in wild or crop hosts. Furthermore, the symptoms were rarely specific to any of the viruses as we only found a significant positive association between infection by PlLV and yellowing. Proving a causal relationship between viruses and the symptoms is particularly challenging as the symptoms may be modified under certain environmental conditions, or stressed plants may be generally more susceptible for the viruses or their vectors (Alexander et al., 2014). It should be noted that other yet-unknown viruses may have been present in these plants and causing the observed symptoms. Furthermore, in our laboratory experiment the tested plants did not show conspicuous symptoms indicating that these viruses also occur as latent infections. Hence, the onset of symptoms may be context dependent.

We tested different inoculation methods for future experimental studies. We found that PlLV can be transmitted using rosy apple aphid (*D. plantaginea*) as a vector. This is in line with a recent study on *Alfalfa leaf curl virus* that reported aphid transmission for the first time for geminiviruses (Roumagnac et al., 2015). We also found that three of the viruses studied can be transmitted using sap from plant to plant. Development of virus inoculation and maintenance protocol is an important step in establishing an experimental system for pathogen studies. When doing the experiments, we used inoculation material that tested positive for a given virus with (RT-)PCR but we did not measure the actual virus load in the plant. Further development of the inoculation protocol requires defining the optimal cycle for transmissions when the virus load is high in the source plants at the time of transmission. Besides variation in virus load, the differences in inoculation success may depend on variation in host range of the viruses. Moreover, while the other four virus taxa have been reported to be transmitted by invertebrate vectors, the transmission of partitiviruses may occur only in the germ line or via fungal pathogens (Fauquet et al., 2012).

Plant pathology research has largely focused on viruses infecting crop species and thus very little is known on the viruses present in wild plant populations (Alexander et al., 2014; Bernardo et al., 2017). This is surprising given that viruses have a long evolutionary history with wild plants before domestication (Burdon, 1996), and viruses may move from wild to cultivated hosts and vice versa (Alexander et al., 2014). The movement between wild and cultivated hosts is expected to have consequences for pathogen evolution and epidemiology (Alexander et al., 2014; Papaix, Burdon & Thrall, 2015). Interestingly, the putative novel viruses found in *P. lanceolata* populations are assigned to genera typically showing wide host range as well as transmission by generalist vectors, e.g., caulimoviruses*,* closteroviruses and enamoviruses. Previously it has also been shown that luteoviruses and geminiviruses can move between wild and cultivated hosts (Garcia-Andres et al., 2006; Power et al., 2011). *Plantago lanceolata* populations often occur adjacent to agricultural fields, and it is a host plant for several generalist herbivores (Nieminen & Vikberg, 2015). Utilizing the primers developed in this study to screen virus prevalence in other wild and cultivated hosts opens new avenues of research on the epidemiology and evolution of viruses at the agro-ecological interface.

Our results increase understanding of the pathogen diversity present in wild populations—local virus communities are diverse and spatially variable. The observed low similarity with previously characterized viruses underlines the need for more studies on natural ecosystems to uncover true levels of pathogen diversity. Coinfections are proving to be common in both natural and agricultural environments (Tollenaere, Susi & Laine, 2016), and our finding of frequent virus coinfections further emphasises the need to study the full within host pathogen diversity. In conclusion, establishing direct links between naturally occurring virus communities, relevant environmental characteristics, and host resistance offers an exciting avenue of future research and has the potential to yield ground breaking insight in to problems at the heart of disease biology: Can we predict disease emergence at the agro-ecological interface (Papaix, Burdon & Thrall, 2015), and what is the role of pathogens in maintaining plant species diversity in natural populations (Bever, Mangan & Alexander, 2015; Lacroix et al., 2014).

##  Supplemental Information

10.7717/peerj.6140/supp-1 Table S1 Contigs with detectable similarity to plant-associated viruses found from 156 bulked plant samples from the Åland Islands in 2013

10.7717/peerj.6140/supp-2 Supplemental Information 1 Virus detections in *Plantago lanceolata* populations in the Åland Islands in 2013 and 2015

## Additional Information and Declarations

### Field Study Permissions

The following information was supplied relating to field study approvals (i.e., approving body and any reference numbers): A specific permit for sampling leaves and seed of *P. lanceolata* was not needed because the plant is not protected species and Finnish legislation (“Jokamiehenoikeus”) allows the sampling of wild species to everyone.

### DNA Deposition

The following information was supplied regarding the deposition of DNA sequences: GenBank accession numbers: MH397358–MH397361.

### Data Availability

The following information was supplied regarding data availability: The raw data on virus detections is available as a Supplemental File.

### New Species Registration

The following information was supplied regarding the registration of a newly described species: Plantago betapartitivirus. This proposed new species [Plantago betapartitivirus] has been submitted to the ICTV for consideration. This name is only valid and official after the ICTV has approved the name, and it has been ratified by the membership: [URL pending]. Plantago latent caulimovirus. This proposed new species [Plantago latent caulimovirus] has been submitted to the ICTV for consideration. This name is only valid and official after the ICTV has approved the name, and it has been ratified by the membership: [URL pending]. Plantago enamovirus. This proposed new species [Plantago enamovirus] has been submitted to the ICTV for consideration. This name is only valid and official after the ICTV has approved the name, and it has been ratified by the membership: [URL pending]. Plantago closterovirus. This proposed new species [Plantago closterovirus] has been submitted to the ICTV for consideration. This name is only valid and official after the ICTV has approved the name, and it has been ratified by the membership: [URL pending].
