# Supplementary material for: Diverse and variable virus communities in wild plant populations revealed by metagenomic tools
Source: PeerJ. 2019 Jan 11;7:e6140. doi: 10.7717/peerj.6140 (PMC6330959; doi:10.7717/peerj.6140)
Supplement: Table S1 [file peerj-07-6140-s001.docx]

| OTU | Family | Genus | Organism | Coding region-related sequence | Population | Sequence length | % of identical matches |
| --- | --- | --- | --- | --- | --- | --- | --- |
| 1 | Caulimoviridae | Caulimovirus | Angelica bushy stunt virus | coat protein | 3375 | 57 | 88.89 |
| 1 |  |  | Atractylodes mild mottle virus | ORF5 | 3375 | 78 | 76.92 |
| 1 |  |  | Atractylodes mild mottle virus | ORF5 | 1719 | 206 | 76.74 |
| 1 |  |  | Atractylodes mild mottle virus | ORF5 | 3178 | 65 | 85.71 |
| 1 |  |  | Carnation etched ring virus | polyprotein | 433 | 75 | 79.17 |
| 1 |  |  | Carnation etched ring virus | polyprotein | 433 | 118 | 78.79 |
| 1 |  |  | Carnation etched ring virus | Coat protein | 1719 | 261 | 51.32 |
| 1 |  |  | Carnation etched ring virus | coat protein, partial | 1719 | 140 | 70.73 |
| 1 |  |  | Carnation etched ring virus | polyprotein, partial | 1719 | 112 | 81.82 |
| 1 |  |  | Carnation etched ring virus | RdRp, partial | 1719 | 72 | 95.65 |
| 1 |  |  | Carnation etched ring virus | RdRp, partial | 1719 | 264 | 81.61 |
| 1 |  |  | Carnation etched ring virus | polyprotein | 3178 | 78 | 80.00 |
| 1 |  |  | Carnation etched ring virus | polyprotein, partial | 3178 | 62 | 100.00 |
| 1 |  |  | Carnation etched ring virus | RdRp, partial | 3178 | 126 | 72.97 |
| 1 |  |  | Cauliflower mosaic virus | reverse transcriptase | 433 | 94 | 74.19 |
| 1 |  |  | Cauliflower mosaic virus | reverse transcriptase, partial | 433 | 63 | 85.00 |
| 1 |  |  | Cauliflower mosaic virus | reverse transcriptase, partial | 1717 | 89 | 88.46 |
| 1 |  |  | Cauliflower mosaic virus | capsid protein | 1719 | 266 | 44.57 |
| 1 |  |  | Cauliflower mosaic virus | movement protein | 1719 | 141 | 59.46 |
| 1 |  |  | Cauliflower mosaic virus | movement protein | 1719 | 111 | 77.78 |
| 1 |  |  | Cauliflower mosaic virus | RdRp, partial | 1719 | 73 | 100.00 |
| 1 |  |  | Cauliflower mosaic virus | reverse transcriptase, partial | 1719 | 77 | 90.48 |
| 1 |  |  | Cauliflower mosaic virus | reverse transcriptase, partial | 1719 | 121 | 63.89 |
| 1 |  |  | Cauliflower mosaic virus | reverse transcriptase, partial | 1719 | 81 | 76.92 |
| 1 |  |  | Cauliflower mosaic virus | reverse transcriptase, partial | 3301 | 71 | 81.82 |
| 1 |  |  | Cauliflower mosaic virus | reverse transcriptase | 3178 | 109 | 72.22 |
| 1 |  |  | Cauliflower mosaic virus (STRAIN BBC) | Cell-to-cell transport protein | 1719 | 114 | 67.86 |
| 1 |  |  | Dahlia common mosaic virus | polymerase polyprotein | 433 | 62 | 95.00 |
| 1 |  |  | Dahlia mosaic virus | polyprotein | 1719 | 91 | 86.67 |
| 1 |  |  | Figwort mosaic virus | unnamed protein product | 45 | 85 | 80.77 |
| 1 |  |  | Figwort mosaic virus | unnamed protein product | 1045 | 80 | 80.77 |
| 1 |  |  | Figwort mosaic virus | unnamed protein product | 3178 | 85 | 80.77 |
| 1 |  |  | Mirabilis mosaic virus | hypothetical protein | 9205 | 102 | 63.64 |
| 1 |  | unclassified Caulimoviridae | Rudbeckia flower distortion virus | putative enzymatic polyprotein | 1045 | 71 | 82.61 |
| 1 |  |  | Rudbeckia flower distortion virus | putative enzymatic polyprotein | 3178 | 147 | 79.17 |
| 1 |  | Caulimovirus | Soybean Putnam virus | reverse transcriptase | 45 | 60 | 100.00 |
| 1 |  |  | Soybean Putnam virus | reverse transcriptase | 1719 | 72 | 86.96 |
| 1 |  |  | Soybean Putnam virus | reverse transcriptase | 1719 | 79 | 88.46 |
| 1 |  |  | Strawberry vein banding virus | putative reverse transcriptase | 45 | 104 | 65.62 |
| 2 | Closteroviridae | Closterovirus | Beet yellow stunt virus |  | 1719 | 198 | 66.15 |
| 2 |  |  | Beet yellow stunt virus |  | 1719 | 102 | 66.67 |
| 2 |  |  | Beet yellow stunt virus |  | 1719 | 482 | 32.26 |
| 2 |  |  | Beet yellow stunt virus |  | 1719 | 1837 | 46.50 |
| 2 |  |  | Beet yellow stunt virus |  | 1719 | 226 | 38.03 |
| 2 |  |  | Beet yellow stunt virus |  | 1719 | 276 | 53.49 |
| 2 |  |  | Beet yellow stunt virus |  | 1719 | 183 | 45.00 |
| 2 |  |  | Beet yellow stunt virus |  | 1719 | 138 | 71.05 |
| 2 |  |  | Beet yellow stunt virus |  | 1719 | 584 | 58.03 |
| 2 |  |  | Beet yellow stunt virus |  | 1719 | 314 | 50.51 |
| 2 |  |  | Beet yellow stunt virus |  | 1719 | 164 | 72.73 |
| 2 |  |  | Beet yellow stunt virus |  | 1719 | 144 | 51.16 |
| 2 |  |  | Beet yellow stunt virus |  | 1719 | 595 | 77.16 |
| 2 |  |  | Beet yellow stunt virus |  | 1719 | 111 | 86.49 |
| 2 |  |  | Beet yellow stunt virus |  | 1719 | 91 | 86.21 |
| 2 |  |  | Beet yellows virus |  | 1719 | 167 | 41.07 |
| 2 |  |  | Beet yellows virus |  | 1719 | 329 | 59.81 |
| 2 |  |  | Beet yellows virus |  | 1719 | 278 | 52.75 |
| 2 |  |  | Beet yellows virus |  | 1719 | 155 | 73.47 |
| 2 |  |  | Beet yellows virus |  | 1719 | 487 | 56.52 |
| 2 |  |  | Beet yellows virus |  | 1719 | 141 | 66.67 |
| 2 |  |  | Beet yellows virus |  | 1719 | 166 | 75.61 |
| 2 |  |  | Beet yellows virus |  | 1719 | 148 | 79.59 |
| 2 |  |  | Grapevine leafroll-associated virus 2 |  | 1719 | 244 | 75.86 |
| 2 |  |  | Grapevine leafroll-associated virus 2 |  | 1719 | 76 | 91.30 |
| 2 |  |  | Grapevine leafroll-associated virus 2 |  | 1719 | 812 | 42.80 |
| 2 |  |  | Grapevine leafroll-associated virus 2 |  | 1719 | 505 | 68.13 |
| 2 |  |  | Grapevine leafroll-associated virus 2 |  | 3178 | 115 | 61.11 |
| 2 |  |  | Mint virus 1 |  | 3178 | 108 | 82.14 |
| 2 |  |  | Raspberry leaf mottle virus |  | 1719 | 85 | 66.67 |
| 3 | Endornaviridae | Alphaendornavirus | Bell pepper alphaendornavirus | RNA dependent RNA polymerase | 3375 | 74 | 86.96 |
| 3 |  | unclassified Endornavirus | Ceratobasidium endornavirus E | polyprotein, partial | 9205 | 104 | 58.82 |
| 3 |  |  | Psophocarpus tetragonolobus endornavirus | polyprotein | 3375 | 80 | 69.23 |
| 3 |  |  | Psophocarpus tetragonolobus endornavirus | polyprotein | 3375 | 75 | 87.50 |
| 3 |  |  | Psophocarpus tetragonolobus endornavirus | polyprotein | 3375 | 155 | 72.00 |
| 3 |  |  | Psophocarpus tetragonolobus endornavirus | polyprotein | 3375 | 178 | 62.71 |
| 3 |  |  | Psophocarpus tetragonolobus endornavirus | polyprotein | 3375 | 161 | 47.17 |
| 3 |  |  | Psophocarpus tetragonolobus endornavirus | polyprotein | 3375 | 65 | 85.71 |
| 3 |  |  | Psophocarpus tetragonolobus endornavirus | polyprotein | 3375 | 132 | 57.14 |
| 3 |  |  | Psophocarpus tetragonolobus endornavirus | polyprotein | 3375 | 138 | 84.44 |
| 3 |  |  | Psophocarpus tetragonolobus endornavirus | polyprotein | 3375 | 130 | 51.16 |
| 3 |  |  | Psophocarpus tetragonolobus endornavirus | polyprotein | 3375 | 122 | 82.05 |
| 3 |  |  | Psophocarpus tetragonolobus endornavirus | polyprotein | 3375 | 114 | 60.00 |
| 4 | Geminiviridae | Capulavirus | Euphorbia caput-medusae latent virus | replication associated protein | 1719 | 64 | 100.00 |
| 4 |  |  | Euphorbia caput-medusae latent virus | replication associated protein | 3178 | 83 | 81.82 |
| 4 |  |  | Plantago lanceolata latent virus | C3 | 1719 | 98 | 96.88 |
| 4 |  |  | Plantago lanceolata latent virus | coat protein | 1719 | 68 | 100.00 |
| 4 |  |  | Plantago lanceolata latent virus | coat protein | 1719 | 258 | 100.00 |
| 4 |  |  | Plantago lanceolata latent virus | coat protein | 1719 | 105 | 100.00 |
| 4 |  |  | Plantago lanceolata latent virus | coat protein | 1719 | 98 | 100.00 |
| 4 |  |  | Plantago lanceolata latent virus | RepA | 1719 | 124 | 97.30 |
| 4 |  |  | Plantago lanceolata latent virus | RepA | 1719 | 109 | 89.29 |
| 4 |  |  | Plantago lanceolata latent virus | RepA | 1719 | 87 | 96.43 |
| 4 |  |  | Plantago lanceolata latent virus | replication-associated protein | 1719 | 130 | 85.29 |
| 4 |  |  | Plantago lanceolata latent virus | replication-associated protein | 1719 | 89 | 91.67 |
| 4 |  |  | Plantago lanceolata latent virus | replication-associated protein | 1719 | 81 | 100.00 |
| 4 |  |  | Plantago lanceolata latent virus | V4 | 1719 | 60 | 100.00 |
| 4 |  |  | Plantago lanceolata latent virus | coat protein | 3178 | 56 | 100.00 |
| 4 |  |  | Plantago lanceolata latent virus | coat protein | 3178 | 71 | 100.00 |
| 4 |  |  | Plantago lanceolata latent virus | coat protein | 3178 | 63 | 100.00 |
| 4 |  |  | Plantago lanceolata latent virus | RepA | 3178 | 82 | 100.00 |
| 4 |  |  | Plantago lanceolata latent virus | RepA | 3178 | 75 | 100.00 |
| 4 |  |  | Plantago lanceolata latent virus | RepA | 3178 | 73 | 100.00 |
| 4 |  |  | Plantago lanceolata latent virus | RepA | 3178 | 129 | 100.00 |
| 4 |  |  | Plantago lanceolata latent virus | RepA | 3178 | 69 | 100.00 |
| 4 |  |  | Plantago lanceolata latent virus | replication-associated protein | 3178 | 74 | 95.83 |
| 5 | Luteoviridae | Enamovirus | Grapevine enamovirus-1 |  | 3375 | 130 | 85.71 |
| 5 |  |  | Grapevine enamovirus-1 |  | 3375 | 120 | 66.67 |
| 5 |  |  | Grapevine enamovirus-1 |  | 3375 | 106 | 62.86 |
| 5 |  |  | Grapevine enamovirus-1 |  | 1719 | 102 | 64.52 |
| 5 |  |  | Grapevine enamovirus-1 |  | 1719 | 244 | 58.33 |
| 5 |  |  | Grapevine enamovirus-1 |  | 1719 | 210 | 76.81 |
| 5 |  |  | Grapevine enamovirus-1 |  | 1719 | 103 | 78.79 |
| 5 |  |  | Grapevine enamovirus-1 |  | 1719 | 105 | 82.86 |
| 5 |  | Polerovirus | Lettuce mild yellows virus |  | 1719 | 106 | 62.86 |
| 6 | Ophioviridae | Ophiovirus | Citrus psorosis ophiovirus |  | 1045 | 67 | 90.48 |
| 6 |  |  | Citrus psorosis virus |  | 9623 | 216 | 44.44 |
| 7 | Partitiviridae | unclassifiedPartitiviridae | Alphacryptovirus JF-2012 | RNA-dependent RNA polymerase | 1045 | 83 | 80.00 |
| 7 |  | Betapartitivirus | Cannabis cryptic virus | RNA-dependent RNA polymerase | 415 | 71 | 86.36 |
| 7 |  | unclassifiedPartitiviridae | Citrullus lanatus cryptic virus | putative RNA-dependent RNA polymerase | 1045 | 92 | 76.67 |
| 7 |  | Betapartitivirus | Crimson clover cryptic virus 2 | RNA-dependent RNA polymerase | 433 | 64 | 100.00 |
| 7 |  |  | Crimson clover cryptic virus 2 | RNA-dependent RNA polymerase | 3301 | 87 | 100.00 |
| 7 |  |  | Dill cryptic virus 2 | RNA-dependent RNA polymerase | 415 | 127 | 84.62 |
| 7 |  |  | Dill cryptic virus 2 | RNA-dependent RNA polymerase | 415 | 110 | 86.67 |
| 7 |  |  | Dill cryptic virus 2 | RNA-dependent RNA polymerase | 415 | 98 | 81.25 |
| 7 |  |  | Dill cryptic virus 2 | RNA-dependent RNA polymerase | 416 | 74 | 83.33 |
| 7 |  |  | Dill cryptic virus 2 | RNA-dependent RNA polymerase | 433 | 100 | 82.76 |
| 7 |  |  | Dill cryptic virus 2 | RNA-dependent RNA polymerase | 1717 | 64 | 95.00 |
| 7 |  |  | Dill cryptic virus 2 | RNA-dependent RNA polymerase | 1717 | 85 | 88.00 |
| 7 |  |  | Dill cryptic virus 2 | coat protein | 3301 | 70 | 100.00 |
| 7 |  |  | Dill cryptic virus 2 | RNA-dependent RNA polymerase | 3301 | 116 | 83.33 |
| 7 |  |  | Dill cryptic virus 2 | RNA-dependent RNA polymerase | 3301 | 80 | 96.15 |
| 7 |  | Deltapartitivirus | Medicago sativa deltapartitivirus 1 | RNA-dependent RNA polymerase | 1045 | 107 | 94.29 |
| 7 |  |  | Medicago sativa deltapartitivirus 1 | RNA-dependent RNA polymerase | 1045 | 79 | 80.00 |
| 7 |  |  | Pepper cryptic virus 1 | RNA-dependent RNA polymerase-like protein | 1045 | 89 | 84.62 |
| 7 |  | unclassifiedPartitiviridae | Persimmon cryptic virus | RNA dependent RNA polymerase, partial | 1717 | 99 | 75.76 |
| 7 |  |  | Persimmon cryptic virus | coat protein | 1719 | 104 | 52.94 |
| 7 |  | Betapartitivirus | Red clover cryptic virus 2 | RNA-dependent RNA polymerase | 433 | 123 | 80.00 |
| 7 |  |  | Red clover cryptic virus 2 | RNA-dependent RNA polymerase | 1717 | 116 | 75.00 |
| 7 |  |  | Red clover cryptic virus 2 | RNA-dependent RNA polymerase | 1717 | 97 | 84.38 |
| 7 |  |  | Red clover cryptic virus 2 | RNA-dependent RNA polymerase | 3301 | 105 | 76.47 |
| 7 |  | unclassifiedPartitiviridae | Sinapis alba cryptic virus 1 | RNA dependent RNA polymerase | 1045 | 226 | 57.33 |
| 7 |  | Betapartitivirus | White clover cryptic virus 2 | RNA-dependent RNA polymerase | 3301 | 76 | 84.00 |
| 7 |  |  | White clover cryptic virus 2 | RNA-dependent RNA polymerase | 3301 | 64 | 100.00 |
| 8 | unclassified viruses |  | Pyrus pyrifolia cryptic virus | capsid protein | 433 | 79 | 65.22 |
| 8 | unclassified viruses |  | Pyrus pyrifolia cryptic virus | capsid protein | 1045 | 96 | 75.00 |
| 8 | unclassified viruses |  | Pyrus pyrifolia cryptic virus | capsid protein | 1717 | 90 | 75.00 |
| 9 | unclassified RNA viruses |  | Apple rubbery wood virus 2 | capsid protein | 1719 | 362 | 31.68 |
